# A Network-Based Approach on Elucidating the Multi-Faceted Nature of Chronological Aging in *S. cerevisiae*

**DOI:** 10.1371/journal.pone.0029284

**Published:** 2011-12-21

**Authors:** Esra Borklu Yucel, Kutlu O. Ulgen

**Affiliations:** Department of Chemical Engineering, Bogazici University, Istanbul, Turkey; University of South Florida College of Medicine, United States of America

## Abstract

**Background:**

Cellular mechanisms leading to aging and therefore increasing susceptibility to age-related diseases are a central topic of research since aging is the ultimate, yet not understood mechanism of the fate of a cell. Studies with model organisms have been conducted to ellucidate these mechanisms, and chronological aging of yeast has been extensively used as a model for oxidative stress and aging of postmitotic tissues in higher eukaryotes.

**Methodology/Principal Findings:**

The chronological aging network of yeast was reconstructed by integrating protein-protein interaction data with gene ontology terms. The reconstructed network was then statistically “tuned” based on the betweenness centrality values of the nodes to compensate for the computer automated method. Both the originally reconstructed and tuned networks were subjected to topological and modular analyses. Finally, an ultimate “heart” network was obtained via pooling the step specific key proteins, which resulted from the decomposition of the linear paths depicting several signaling routes in the tuned network.

**Conclusions/Significance:**

The reconstructed networks are of scale-free and hierarchical nature, following a power law model with γ  =  1.49. The results of modular and topological analyses verified that the tuning method was successful. The significantly enriched gene ontology terms of the modular analysis confirmed also that the multifactorial nature of chronological aging was captured by the tuned network. The interplay between various signaling pathways such as TOR, Akt/PKB and cAMP/Protein kinase A was summarized in the “heart” network originated from linear path analysis. The deletion of four genes, *TCB3*, *SNA3*, *PST2* and *YGR130C,* was found to increase the chronological life span of yeast. The reconstructed networks can also give insight about the effect of other cellular machineries on chronological aging by targeting different signaling pathways in the linear path analysis, along with unraveling of novel proteins playing part in these pathways.

## Introduction

Aging is usually defined as the progressive loss of function accompanied by decreasing fertility and increasing mortality with advancing time, due to the accumulation of molecular, cellular and organ damage. Although it is clear and evident that aging “occurs”, the reasons, pathways and regulators responsible for the mentioned accumulation of deleterious effects are still vaguely described, rendering the mechanisms that contribute to aging and age-associated diseases a central topic of interest. Recent works on model organisms such as yeast, worms and flies have yielded promising discoveries regarding these mechanisms [1], [2] which may be projected to higher eukaryotes. The yeast *Saccharomyces cerevisiae*, an extensively used model organism, harbors two models of aging: Replicative and Chronological Aging. Replicative aging term is used for the aging of mitotically active yeast cells, involving the capacity of daughter cell production of a mother cell, before senescence [3]. However, yeast chronological life span is the length of time a population remains viable in the non-dividing, quiescent state [4], which is thought to be a suitable model for aging of post-mitotic tissues [5]. Chronologically aged yeast cultures die exhibiting typical markers of apoptosis, accumulate oxygen radicals, and show caspase activation [6], i.e. processes crucial for the cell fate of other higher eukaryotes. Several alterations in signaling pathways such as TOR, Akt/PKB and cAMP/Protein kinase A, which are also conserved between yeast and higher eukaryotes such as *Homo sapiens*, have been demonstrated to affect the damage accumulation previously mentioned [7]–[10]. In yeast, these pathways may be represented by orthologous proteins like Tor1p, Sch9p and Ras2p respectively. These points altogether, render chronological aging machinery of yeast as a promising candidate for gaining insight about aging and age-related diseases in humans.

Recently, research has been conducted to comprehend connectivity between longevity and age-related diseases along with the determination of genes regulating life span, using systems biology approaches [11]–[18]. Almost all of the stated studies benefit from published protein-protein interaction (PPI) data to construct a biological network, which is then topologically analyzed. Studies investigating aging and age-related diseases in humans employ different topological techniques, such as shortest path length [7], [14] and connectivity [7], [11], [12], [14], [16], [18] analyses on the reconstructed PPI networks. Also, the integration of intracellular PPI data with extracellular ones is another approach in network reconstruction employed by human aging studies [13]. The networks of individual signaling pathways affecting aging, such as TOR pathway [8] and glucose repression pathway [15], are important examples of network based approaches in elucidating aging process. In *S. cerevisiae*, the two aging processes encountered have also been subjected to network-based analysis. The application of shortest path length analysis on a longevity network constructed with PPI data of proteins related to replicative aging process [19], and topological analysis of a hybrid aging network, reconstructed by integrating both replicative and chronological aging processes, [20] gave information about novel genes and processes which impact both types of aging in yeast. Moreover, examples of the “bottom-up” systems biology which involve the construction of an *in silico* model with genes, proteins and processes as parameters have also been encountered while investigating aging in yeast [17] and in higher eukaryotes [21].

In the current study, Chronological Aging Network of *S. cerevisiae* is reconstructed using Selective Permissibility Algorithm (SPA) which integrates Gene Ontology (GO) annotation terms with protein-protein interaction (PPI) data, in an automated manner [22]. False positives naturally occurring in PPI data and insignificant PPI's are eliminated from the reconstructed network by statistical methods based on betweenness centrality values, and the tuned network is then clustered and subjected to linear path analysis. Via linear path analysis, routes starting with proteins previously demonstrated to regulate life span such as Tor1p (homologous to mammalian mTOR), Sch9p (homologous to mammalian Akt/PKB) and Ras2p (homologous to mammalian Ras proto-oncogenes) together with 3 other proteins (Gpa2p, Pga3p and Ptk2p) and ending at Sir2p and Gts1p are investigated. Simultaneous analysis of the linear path spectra of these input-output pairs enable one to unravel intermediate players of the signaling events that lead to chronological aging. Step-specific key protein determination is the chosen method in the current study for the mentioned in depth analysis, yielding a denser final network of 92 nodes for the 6 input and 2 output proteins. This dense “heart” network depicts the routes highly participating to the information flow in the network by identifying fundamental proteins for the proceeding of the signal transduction for studied input-output pairs. Indeed, four proteins of this heart network, Tcb3p, Sna3p, Pst2p and YGR130Cp, which have not been reported to affect chronological aging and also have unknown GO process terms, are demonstrated to be involved in life span alteration.

Reconstruction and dissection of the reconstructed network as well as its topological analysis, helps us unravel and enlighten the inner dynamics of chronological aging mechanism of *Saccharomyces cerevisiae.* Only the members of the nutrient sensing pathways (Tor1p, Gpa2p, Ras2p, and Sch9p) with some other input proteins such as Pga3p, which is proved to regulate the life span, and Ptk2p, which is involved in cellular ion homeostasis, are investigated in the current study. Further analyses of linear paths starting with other proteins taking part in different signaling pathways will provide data to illuminate the possible machineries by which the mentioned signaling pathways affect the chronological life span as well as to decipher the important proteins responsible for these effects. The proposed framework can effectively be used as a tool to give insight about other biological networks, regardless of the species of which they belong.

## Results

### Network Reconstruction and Reduction

**Figure 1:**
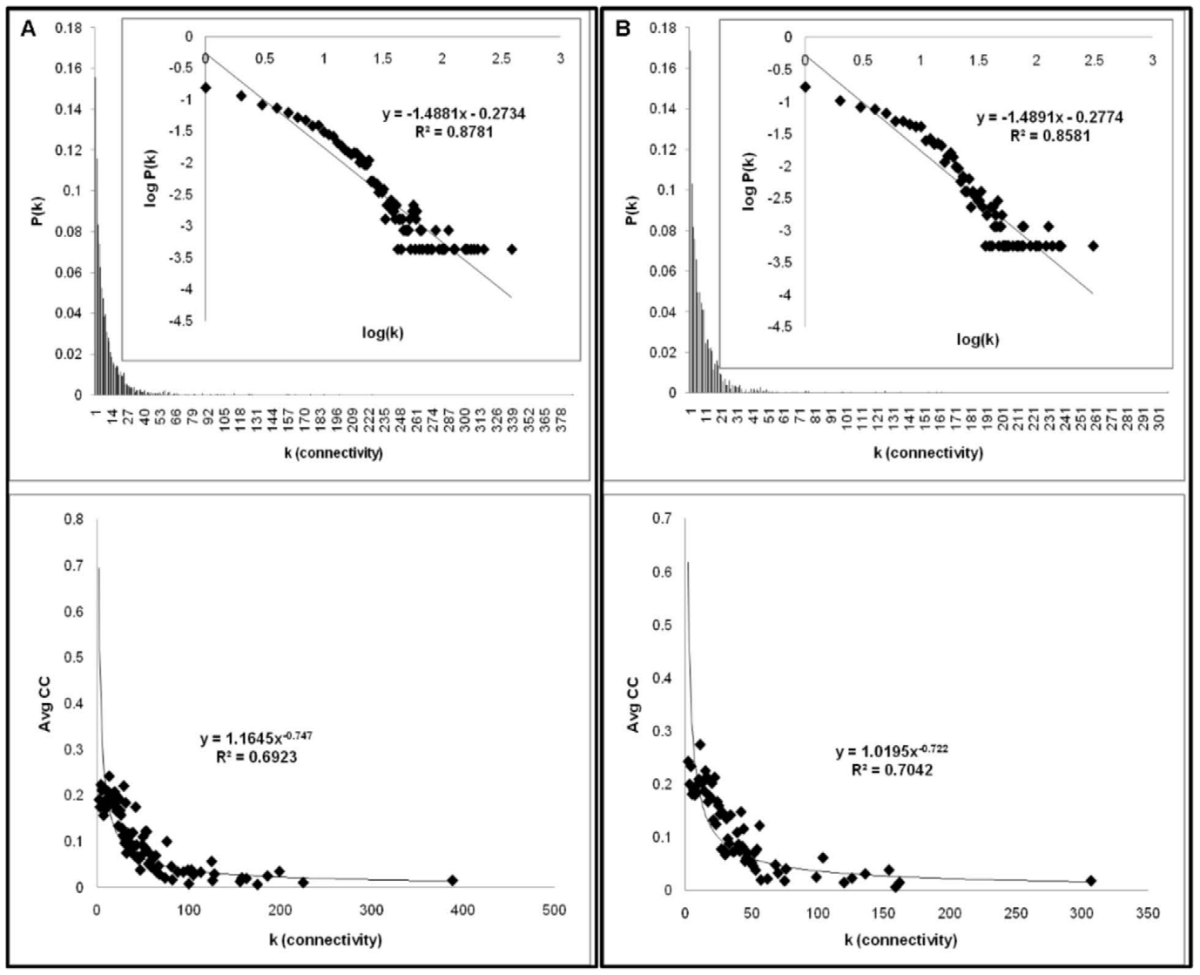
Connectivity and average clustering coefficient distributions for a) CAN and b) tCAN.

In the present study, the ‘‘chronological aging network’’ (CAN) in *Saccharomyces cerevisiae* is reconstructed via integration of protein–protein interactions with Gene Ontology terms. To achieve this goal, all proteins which share the “chronological aging” term under biological process were selected as the core constituents of the network to be reconstructed (Table S1). The network was then expanded as described in the Methods section (Figure S1). The 18 core proteins led to an undirected graph composed of 2359 nodes and 12314 edges as the final network (Table S2). The network diameter and the mean path length are found to be 9 and 3.37 respectively. These distance measures are orders of magnitude significantly smaller than the number of proteins, meaning that despite the large size of the network, any two nodes in the network can be connected by relatively short paths along existing links, emphasizing the small world architecture of the reconstructed network. Moreover, as in many biological networks, the distribution of the nodes in the current reconstructed network has a scale-free nature following nearly a power law model, P(k) ≈k^−γ^, having γ  =  1.49 with R^2^  =  0.88 (Figure 1a). Other topological parameters such as average degree, critical path length, diameter and average clustering coefficient are also in close vicinity with the values reported for other protein-protein interaction networks in literature (Table 1). Further analysis of average clustering coefficient values versus degree reveals that the current system is actually a hierarchical network, resembling the Barabasi-Albert model discussed in elsewhere [23], [24]. The distribution of average clustering coefficients with respect to degree follows a power law model with C(k) ≈k^−w^, having w ≈0.75 with R^2^  =  0.69 (Figure 1a). These topological parameters, γ and w, imply that the network reconstructed is made of numerous small, highly integrated modules, preserving both the high degree of clustering and the scale-free property. In fact, when the same topological analysis is carried out with the network comprised of the whole PPI data in BioGrid (BioGrid network), it is observed that both γ and goodness of fit value, R^2^, decreases. Moreover, the BioGrid network does not follow a hierarchical nature, since w is found to be 0.615 with a considerably small R^2^ value of 0.46 (Figure S2).

**Table 1:** Topological properties of protein-protein interaction networks.

Model	node #	edge #	<k>	CPL	d	<CC>	Reference
CAN (*S. cerevisiae*)	2359	12314	10.4	3.4	9	0.157	this study
tCAN (*S. cerevisiae*)	1736	8458	9.7	3.4	9	0.167	this study
Signaling (*S. cerevisiae)*	1363	3649	5.4	6.8	9	-	[22]
Ca^2+^ signaling (*S. cerevisiae*)	1826	10078	11.04	3.56	11	0.150	[115]
DIP *(M. musculus)*	329	286	-	3.6	9	0.155	[116]
DIP *(H. sapiens)*	1065	1369	-	6.8	21	0.206	[116]
Wnt signaling *(H. sapiens)*	3489	10092	-	4.4	15	-	[117]
EGFR signaling *(H. sapiens)*	329	1795	10.91	4.7	11	-	[95]
Hedgehog Signaling *(D. melanogaster)*	568	975	-	4.8	14	-	[118]
Wnt/β-catenin Signaling *(D. melanogaster)*	656	1253	-	4.8	13	-	[118]

“<k>” denotes the average connectivity, “CPL” stands for critical path length, “d” is diameter and “<CC>” is the average clustering coefficient value.

It is well known that the aging process is a multifactorial phenomenon; i.e. various parameters affect the lifespan of yeast as well as of higher eukaryotes via different branches in cell [12], [25], [18]. Hence it is not a surprising result that the size of the reconstructed network is fairly large, integrating various mechanisms attached to the process of chronological aging of yeast. The current reconstruction of the network was carried out in an automated fashion to eliminate possible biases which may arise from manual curation. But unfortunately, that also renders the network prone to the incorporation of incomplete and/or erroneous data (e.g. false positives) which originate unavoidably when high throughput experiments are carried out. To counterbalance this side-effect of the method, a hypothesis testing based on the betweenness centrality (BC) values of individual nodes was carried out as described in the Methods section. Briefly, it was assumed that if the BC value of a node does not change significantly for both the reconstructed and randomized networks (the average value of 100 networks in the randomized case), the node was considered to be included in the network randomly and therefore discarded, since its contribution to the putative information flow in the random networks is the same as its contribution to the real information flow in the original network. Three different significance levels were employed for this hypothesis testing: 0.1, 0.01 and 0.001. Ultimately the significance level value (α) of 0.001 was selected to be used as the threshold since the resulting network with this significance value yielded the highest goodness of fit score, R^2^  =  0.86, compared to the other two significance level values for the power law model (Figure S3). Following this hypothesis testing, approximately 26% of the original nodes were considered as statistically insignificant and they were filtered out from the originally reconstructed network along with their interactions (31% of the original interactions). Ultimately, the “tuned” network obtained (tCAN) had 1734 nodes and 8458 interactions (Table S3). Moreover, when tCAN was topologically analyzed, it was observed that many of the statistical parameters remained nearly unchanged, compared to those of CAN (Figure 1b, Table 1).

### Hubs and Clusters of CAN and tCAN

#### Hub Proteins

**Figure 2:**
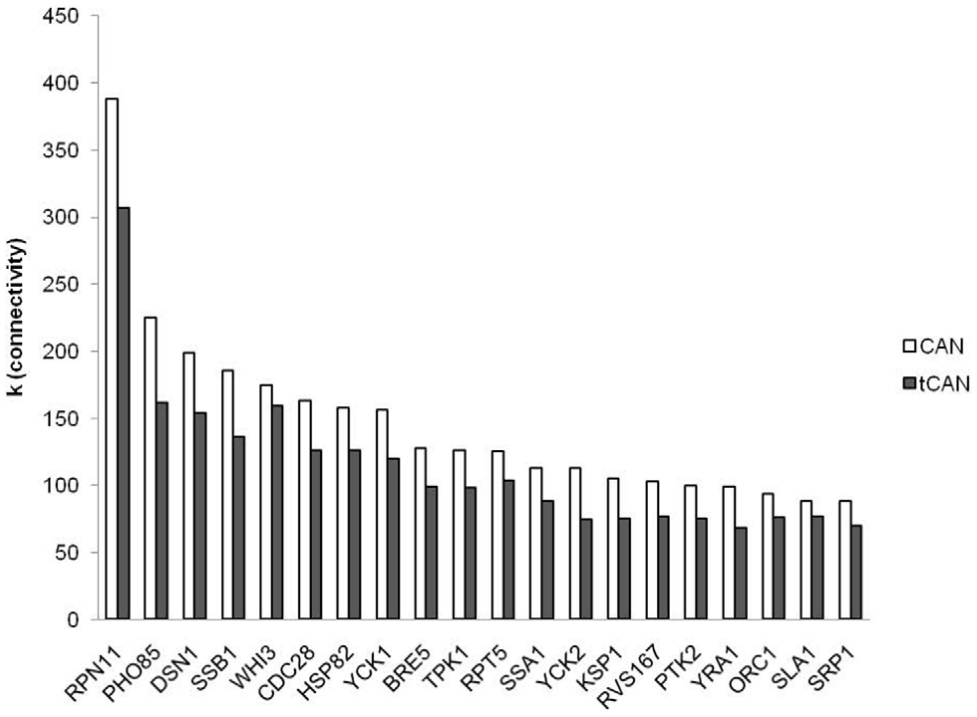
The first 20 hub nodes in the two networks with their corresponding connectivity values.

The first 20 of the highly connected nodes, referred to as the “hubs” of the network, are the same proteins in both CAN and tCAN, although their ranking differed slightly for the two networks (Figure 2). 13 of these hubs are also among the first 20 hubs of BioGrid network; however their specificity in CAN differs considerably compared to that in BioGrid network. The individual deletion of the 10 common hubs results in up to 6 and 5 fold increase in the number of connected components in CAN and in tCAN respectively, compared to BioGrid network, except the deletion of *TPK1* (1.33 fold change in BioGrid Network compared to CAN and tCAN), *RPT5* (connected component number is the same for all three networks) and *RPN11* (2.5 fold change in CAN while created connected component number does not change in tCAN, compared to BioGrid Network) (Table S4). This result demonstrates that in CAN, these hub proteins are more important in terms of network stability and robustness: CAN is more prone to “attacks” targeting these hubs than the BioGrid network.

When these 20 hubs are analyzed thoroughly, it is observed that they are indeed strongly related to the chronological aging and quiescence processes in yeast. A very recent genome-wide study demonstrated that the deletion mutants of the genes encoding 6 of these 20 hub proteins had either shorter (*WHI3*, *TPK1*, *RVS167*) or longer (*PHO85*, *BRE5*, *SSA1*) chronological life spans compared to that of wild type strain [26]. Although the effect of the essential gene encoding the hub protein with the highest degree, *RPN11*, on chronological aging of *S. cerevisiae* has not yet been investigated, Tonoki and colleagues demonstrated that the loss of function of *RPN11* resulted in a shorter life span for *D. melanogaster* [27]. Harris et al. proved that reduced levels of Hsp82p activity in *S. cerevisiae* resulted in a longer chronological life span, by increasing the stress resistance of the cells [28].

The remaining 12 hub proteins have not been subject to experimentation yet to determine their effects on the chronological life span of yeast. However, when their roles in the cellular machinery are concerned it is seen that they take part in crucial cellular processes closely linked to aging such as maintenance of genomic stability, actin dynamics, protein degradation and regulation of pH. For instance, Cdc28p was required to generate post senescence survivors at a normal rate in telomerase-negative *S. cerevisiae* cells [29] together with a role in maintenance of genomic stability [30]. The dysfunction of telomeres induces senescence [31] and has also been hardwired with chronological aging in yeast [32], [33]. Rpt5p, another hub protein of the network, was identified to be among the proteins which affect the telomere length in *S. cerevisiae* [31]. The 3^rd^ hub protein of CAN (and 4^th^ of tCAN), Dsn1p, is a member of the MIND kinetochore and is responsible for the accurate segregation of chromosomes [34]; therefore altered Dsn1p activity results in the disruption of genomic stability, which in turn may trigger chronological aging [35]. The mRNA-binding protein Yra1p, another hub of the networks under study, has a role in the DNA damage response of the yeast cells via nucleotide excision repair (NER) system. This repair system was proved to provide protection against both cancer and aging [36], [37]; hence Yra1p may affect the chronological aging mechanism in *S. cerevisiae* by altering the genomic stability.

The three hub proteins Ptk2p, Yck1p and Yck2p are found to be involved in the phosphorylation of Pma1p, a cell surface protein which is the major regulator of cytoplasmic pH and plasma membrane potential [38], [39]. The limiting effect of extracellular acidity on the chronological aging of *S. cerevisiae* is well established [40], therefore these 3 hub proteins may affect the response of the cell to acid toxicity via tampering Pma1p activity. Sla1p, protein required for the assembly of the cortical actin cytoskeleton, is another hub protein of the network. It was suggested that the actin cytoskeleton interacts with the mitochondria, and this interaction plays a significant role in programmed cell death and aging [41]. *SSB1* encodes Ssb1p, a member of the Hsp70 family together with another hub protein, Ssa1p, and is considered to take part in the proper folding of newly synthesized proteins as well as in the glucose sensing pathway in *S. cerevisiae* [42], [43]. Both protein homeostasis [44] and glucose signaling [45] are suggested to be the key factors in aging process. In fact the two hub proteins mentioned previously, Yck1p and Yck2p are also taking parts in Snf3/Rgt2-mediated glucose signaling of yeast [46]. Protein homeostasis includes proper degradation as well as proper folding machinery of proteins, since accumulation of misfolded or damaged proteins leads to protein toxicity and aging. The hub protein Srp1p was demonstrated to be a part of this degradation machinery in *S. cerevisiae* through ubiquitin-proteasome system [47], and may therefore be influential on chronological aging mechanism via maintenance of protein homeostasis in the cell. Ksp1p, a ser/thr protein kinase required for growth in nutrient limited conditions, is another hub protein of the network. It has recently been demonstrated that Ksp1p is amongst the Tor1p-regulated phosphoproteome; much specifically it is one of the rapamycin-sensitive phosphoproteins of yeast coordinated by the Sch9p branch of the TOR signaling [48], [49]. Moreover Ksp1p regulates the translocation of Bcy1p, the regulatory subunit of the cAMP-dependent PKA [50]. Both TOR and cAMP/Protein kinase A signaling pathways are reported to be responsible for life extension during calorie restriction in yeast, hence it is not surprising to detect Ksp1p as a hub protein in CAN and tCAN [51]. The last one of the 20 hub proteins of CAN and tCAN is Orc1p, the largest subunit of the origin recognition complex of yeast. Orc1p is the paralog of Sir3p [52], the silencing protein required for spreading of silenced chromatin. Although the sequences of *ORC1* and *SIR3* have diverged significantly, Orc1p is also involved in transcriptional silencing, rendering itself as a suitable hub protein of CAN and tCAN since gene silencing leads to cellular quiescence [53].

#### Modular Analysis

The reconstructed networks (CAN and tCAN) are of hierarchical nature, whereas the BioGrid network is not, which implies a reigning modular topology within CAN and tCAN. The modules (the highly connected protein subgroups) are expected to give insight about underlying cellular machineries leading to chronological aging process in yeast (Tables S5 and S6). Actually, when modularity analysis is performed to the whole PPI data in BioGrid (Table S7), it is observed that only 5% of the proteins encountered in the modules of CAN and tCAN are shared by the protein spectrum of the BioGrid network. Furthermore, the enrichment analyses of the modules of the BioGrid network (having scores greater than 3) result in parent GO terms predominantly, complicating the extraction of differential information from the analysis (Table S8). However, the enriched GO process terms of modules of CAN and tCAN reflect the cellular reprogramming necessary for quiescence which is the hallmark of chronological aging, validating the fact that the reconstructed networks indeed encompass different aspects connected to chronological aging of yeast through this modular topology. The filtered enriched terms for the clusters of CAN and tCAN are summarized in Table S9 and Table S10 respectively.

Many terms related to cell cycle (S phase, interphase etc.) but especially to its mitotic (M) phase (DNA replication initiation, cytoskeleton organization, chromosome segregation and organization, cytokinesis, etc.) stand out among the enriched categories following the modular analysis on the complete set of proteins of CAN and tCAN (see Tables S9 and S10 for p-values). It was surprising to have GO terms related to mitotic cell cycle, since chronologically aged yeast cells are in a quiescent state (G_0_ phase)[54], and do not divide. However, it has recently been reported that along with the increase in genomic instability, a breakdown in mitotic asymmetry is also encountered in chronologically aged yeast cells [55]. This renders the mitotic division mechanism of importance for not solely replicative aging process, but for both types of aging in yeast, coherent with the findings of this study. Apart from cell cycle terms, the enriched GO categories for both networks may be gathered into three main cellular processes which are required to maintain viability in the quiescent state: i) reorganization of metabolism, ii) redox homeostasis and iii) protein turnover.

#### Reorganization of metabolism

The trehalose and vitamin B6 (pyridoxine) biosynthesis, which are the most significantly enriched terms of the two clusters (15 & 16) in both networks (p-values<10E-5), are examples of the metabolic rearrangement encountered in quiescent cells. *SNZ1*, the product of which is required for pyridoxine biosynthesis, is identified to be expressed after entry into quiescence [56], and then a role of vitamin B6 as a cofactor in stationary-phase specific processes or as an antioxidant has been suggested [57]. Similarly, trehalose was proposed to protect proteins against oxidative damage in quiescent cells [58] and moreover, cells metabolize trehalose also for fuel upon exit from the quiescent state [59]. The GO process terms belonging to glycolysis and gluconeogenesis along with respiration and fermentation stood out in the enrichment results (p-values<2E-2). Apart from the tight regulation of these processes in quiescent cells, alternative energy production routes are well activated in chronologically aging cells. The terms “fatty acid metabolism” and “triacylglycerol mobilization” are among the enriched categories in this study, indicating that the networks reconstructed contained the other speculated energy source of quiescent cells: triacylglycerol and/or fatty acid oxidation [60]. The possibility of deriving energy from fatty acid catabolism implies an important role for peroxisome and mitochondria in the maintenance of a quiescent cell. In fact many terms related to the mitochondrial as well as peroxisomal functions appear in the enrichment results of tCAN (and similarly of CAN), in accordance with the literature [61], [62]. The terms “cell wall organization and biogenesis”, “cell wall chitin metabolic process” and “ergosterol biosynthetic process” give insight about another aspect of the metabolic reorganization encountered in quiescence, the remodeling of the cell wall. Actually, quiescent cells develop thickened cell walls [63] and that cell wall organization in yeast cells is highly dependent on PKA signaling pathway which may negatively regulate longevity in yeast [64]. Moreover, ergosterol, which is an essential lipid for the membrane, is taking role in the response to oxidative stress and is shown to be a part of the longevity network of yeast [65], [17].

#### Redox homeostasis

The reorganization of energy metabolism in quiescent cells is closely linked to the redox homeostasis machinery. As mentioned above, the storage of carbohydrates such as trehalose and glycogen together with hydrolysis of lipid stores in quiescent cells probably leads to the accumulation of free fatty acids. Peroxisomes come onto stage at this point, and oxidize these fatty acids to acetyl-CoA, which is subsequently oxidized in mitochondria to generate ATP in quiescent cells [60]. The terms involving the electron transport chain along with “TCA cycle” and “Acetyl-CoA catabolism” terms in the enrichment results also support this hypothesis. However, this reorganization in the cell generates considerable quantities of mitochondrial ROS and oxidative stress, pointed out by the enriched “superoxide metabolic process”, “oxygen and reactive oxygen species metabolic process”, and “age-dependent response to reactive oxygen species during chronological cell aging” terms in GO enrichment analysis. In fact, ROS homeostasis must be tightly regulated in quiescent cells, since it is debated that ROS play a dual role in determining the fate of a cell [66]: ROS are speculated to increase the chronological lifespan of the whole culture, below a certain threshold, probably by triggering autophagy [67], [68] and/or apoptosis [69], [6] but they may very well decrease it, via exactly the same mechanisms, likely when their level exceeds the threshold [70]–[72]. Coherent with the mentioned points above, the apoptosis and autophagy related terms are also present in the enrichment results of both networks.

#### Protein turnover

The most significant GO process term for both networks is “ubiquitin-dependent protein catabolic process”, having the highest corrected p-value. Ubiquitin/proteasome system is one of the protein turnover mechanisms in yeast and was demonstrated to contribute to chronological aging in yeast [19], [73]. Whether the ubiquitin dependent protein degradation is a mechanism necessary for oxidative stress resistance of yeast cells is a highly debated issue. Recently, however, it was demonstrated that ubiquitin, as well as proteasome, is necessary for oxidative stress resistance [74]. This information relates the above mentioned ROS homeostasis and protein turnover machineries in quiescent cells: proper degradation of oxidized proteins resulting from accumulated ROS may be necessary for maintaining viability in quiescent state. Moreover, autophagy and ubiquitin/proteasome system have been proved to be cross-linked [75], hence this mechanism may also be effective in the amino acid recycling of stationary phase yeast cells along with autophagy, which has been proved to affect the chronological life span of *S. cerevisiae*. “Protein amino acid deacetylation” is another term that comes across the results of this study concerning protein modification, implying its importance for quiescent cells. Indeed, studies adopting spermidine and resveratrol, both of which are known to extend the chronological life span of yeast by inducing autophagy, have hinted that these agents activate the autophagic cascade in the cell via deacetylation reactions [76], [77]. The majority of the remaining enriched GO terms are related to intracellular (especially vesicle-mediated) transport involving endosomes, membrane invagination, endocytosis, exocytosis, and actin cytoskeleton organization. These processes are also closely linked to the autophagy machinery which promotes the survival of quiescent cells [78]–[80].

Considering the similarities in the topological properties as well as in the enrichment results of CAN and tCAN, it was decided to adopt tCAN in the forthcoming analyses.

### Linear Path and Key Protein Analysis

#### Linear Path Analysis

By investigating the linear paths between particular proteins in tCAN it is aimed to gain insight about the information flow of the intracellular machineries leading to chronological aging in yeast. Six input (Pga3p, Tor1p, Gpa2p, Ptk2p, Ras2p and Sch9p) and two output (Sir2p and Gts1p) proteins were selected for linear path analysis. The role of Tor1p and Ras2p, the two membrane proteins, is well established in chronological aging [4], [81]. Pga3p is a putative cytochrome b5 reductase on the plasma membrane, and one of the core proteins of the reconstructed network [82]. *GPA2* encodes a subunit of the heterotrimeric G protein that interacts with the receptor Gpr1p, which has a signaling role in response to glucose and its deletion mutant has been demonstrated to have a longer chronological life span compared to wild type strain [83]. Ptk2p regulates the ion transport across plasma membrane and enhances spermidine uptake [84]. Moreover it is one of the first 20 hub proteins of the network. The involvement of Sch9p in the regulation of life span in yeast is well documented [85], but it is not localized to the plasma membrane. The two end proteins, Sir2p and Gts1p, are among the core proteins of the network, and both have a transcription regulator activity. By decomposing tCAN into linear paths, depicting the signaling routes between mentioned inputs and outputs, the intermediate proteins taking part in the embedded information flow can be unraveled.

Using NetSearch algorithm [86], different path lengths starting from 3 to 8 were tested to reach a transcriptional regulator from an input protein and it was seen that as the linear path length increases, the number of paths to be analyzed increased in an exponential fashion (unpublished data). Furthermore, when the number of steps is greater than 7, the data analysis becomes computationally tedious, since the final number of linear paths for a given couple of proteins is in the order of 10^6^ for this number of steps. So, the path length is chosen to be 6 for this study, with an acceptable core and network protein coverage value of 65% and 55% respectively. Alternatively, the same analysis was carried out for Tor1p-Sir2p pair in BioGrid network and it was observed that the number of linear paths increased almost 35 fold (729960 linear paths) while the number of proteins involved in these linear paths increased about 5 fold (3479 proteins) when compared to tCAN. Actually, the total number of proteins taking part in the interaction data of BioGrid release 3.1.73 is 5626, and 61% of these proteins appeared in the results of the linear path analysis for this branch only. Moreover, the protein set obtained by the analysis performed only on Tor1p-Sir2p pair in BioGrid network has a strong similarity to complete tCAN, having 1200 proteins in common. Therefore, it is not surprising to have an increased core protein coverage and percentage of common network proteins, 86% and 69% respectively, for a path length of 6 in BioGrid network for Tor1p-Sir2p branch (unpublished data).

Linear path analysis supplies analytical measures to distinguish the relative activity of the signaling routes under study, aside from pointing out candidate proteins belonging to a signaling cascade. As a general result, it can be deduced that the Sir2p branch of the pathway is more active compared to Gts1p branch, since an approximately 2-fold increase is observed in the number of linear paths for all input proteins (Table 2). When the input proteins are ranked according to the abundance of linear paths, Ptk2p and Tor1p are the most active ones, followed by Gpa2p. Sch9p and Ras2p, clustered as the third active input proteins, pursuit Gpa2p and finally the least active input protein is Pga3p for both outputs with less than 100 linear paths. The activity of the input proteins may hint to the robustness and therefore to the complexity of the cellular machineries in which these proteins take part. For instance, the higher path numbers belonging to Ptk2p and Tor1p indicate that the signal flowing through these nodes has many alternative routes, implying that these proteins are probably involved in regulating multitudinous complex processes which have an impact on chronological aging. In case a perturbation occurs in one route, there are several alternative routes that may maintain an intact signal transduction if the start proteins are Ptk2p and/or Tor1p [87]. According to this point of view, the signaling cascades involving Pga3p are expected to be very sensitive to any perturbation and should affect the chronological aging in yeast in a relatively direct manner. Indeed, Pga3p is the only membrane protein which is also one of the core proteins of tCAN, and the relatively high percentage of core protein coverage of linear paths starting with Pga3p despite the extremely low total protein coverage implies a relatively direct involvement of this protein in aging [82]. Moreover, all of the linear paths of the Pga3p branch directed to both Sir2p and Gts1p, contain at least one protein which is encoded by a gene whose deletion is lethal to the cell (SGD phenotype data, release 11328), emphasizing the sensitivity of the transduction routes towards perturbations [88].

**Table 2:** The quantitative results of the linear path analysis.

	To Sir2p					To Gts1p				
	# of paths	# of proteins	# of UP's*	CPC** (%)	OPC*** (%)	# of paths	# of proteins	# of UP's*	CPC** (%)	OPC*** (%)
**Ptk2p**	33117	750	15	55.6	43.3	16580	684	106	44.4	39.4
**Tor1p**	21109	689	116	44.4	39.7	10200	651	85	38.9	37.5
**Gpa2p**	9344	510	28	33.3	29.4	4443	485	33	27.8	27.9
**Ras2p**	5591	581	83	38.9	33.5	2500	446	57	38.9	25.7
**Sch9p**	6075	518	35	38.9	29.9	2400	346	3	27.8	19.9
**Pga3p**	73	64	7	22.2	3.7	40	46	5	11.1	2.7

*UP's: Unique proteins, designate proteins solely present in the linear path spectra starting with the mentioned input protein.

**CPC: Core protein coverage, is the percent ratio of the core proteins present in the linear path spectra of the specific input-output pair over those of tCAN.

***OPC: Overall protein coverage, is the percent ratio of the proteins present in the linear path spectra of the specific input-output pair over those of tCAN.

When the proteins involved in linear path spectra are considered, it was noticed that approximately 84% of the proteins involved in Sch9p branch are common with Tor1p branch for Sir2p and this percentage mounts to 95% for the output protein Gts1p. Also, Tor1p branch surpasses Sch9p branch (for all output proteins) in both the unique protein number and linear path number. Hence linear path analysis points to the Sch9p branch to be a sub-cluster of Tor1p branch, and the fact that Sch9p is a substrate of Tor1p supports this finding [89]. The other input protein which has a similar activity (in terms of linear path abundance) with Sch9p is Ras2p. Ras2p and Gpa2p are involved in the transcriptional response to glucose and are members of G-proteins [90], [91]. However, Ras2p branch has a higher unique protein number compared to that of Gpa2p despite a considerably lower linear path number. This fact, when combined with a common protein percentage being lower than 70% (for both Sir2p and Gts1p) between the two branches, indicates that Ras2p branch cannot be considered as a sub-cluster of Gpa2p branch (Table 2). In fact, although both Gpa2p and Ras2p were demonstrated to function similarly in the cell, e.g. to induce cAMP signaling and to mediate the transcriptional response to glucose in yeast [92], they act in redundant pathways, rather than in sequential steps in the same pathway [93], [94].

#### Reconstruction of the heart network

Apart from quantitative analysis, the linear paths of each input-output protein pair were qualitatively investigated to have deeper information about the participations of individual proteins in the information flow. A classical approach for determining the important proteins for an input-output pair is the global investigation of their participation percentage values [95], [96]. Participation percentage value of a protein is the percent ratio of the number of linear paths, in which the mentioned protein is involved, over the total number of linear paths, disregarding the step at which it is contributing to the information flow. Although the important proteins (ranging from 3 to 12 for all pairs) are determined via this method for an input-output pair successfully, they are not always interacting with each other since they do not necessarily emerge from successive steps of a path. But the successive structure of a signaling network, which is crucial in the information flow of biological networks, is enclosed in the currently developed decomposition method (step-specific key protein determination), yielding a more complete spectrum for important proteins, compared to the global percentage analysis.

**Figure 3:**
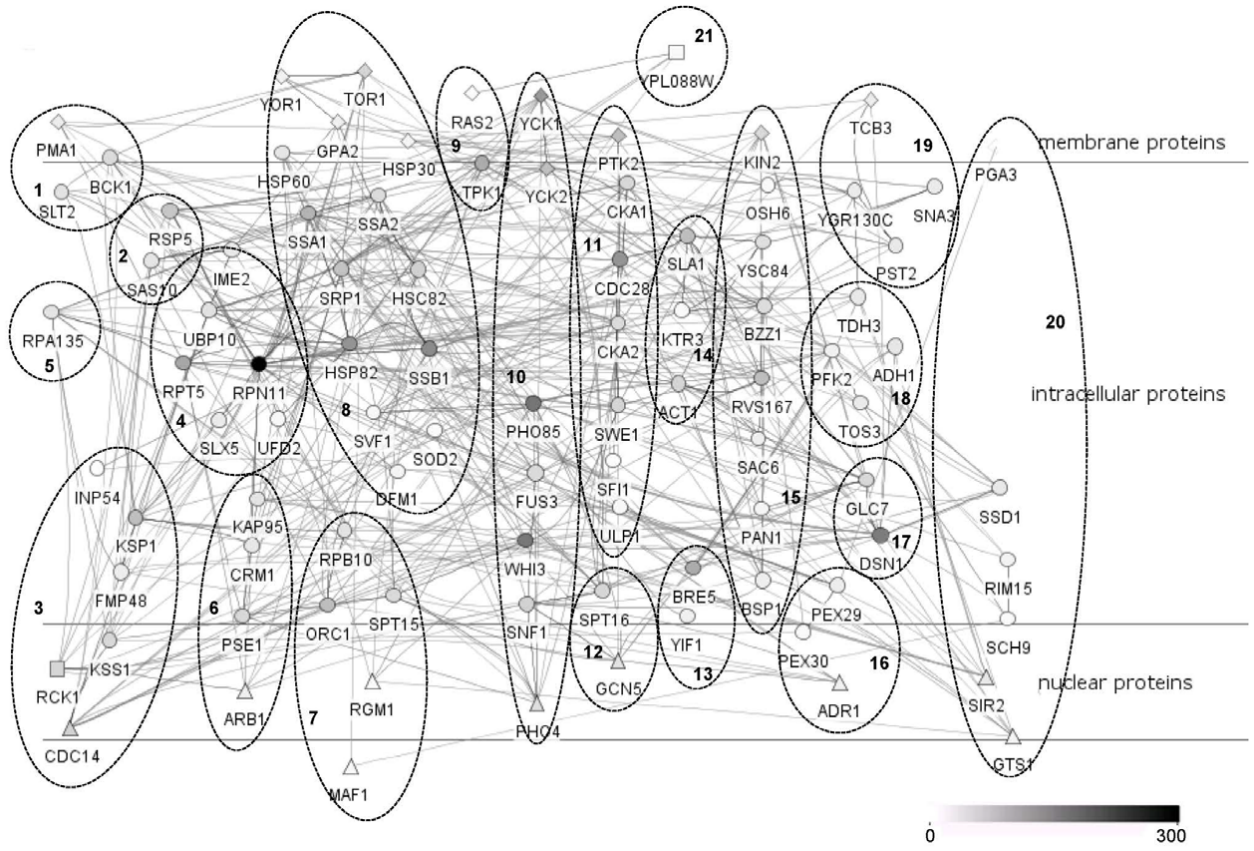
The “heart” network depicting the key proteins of the 12 branches. The node colors reflect the degree of the proteins in tCAN. The GO enrichment results of the numbered clusters are given in Table S4. The circular and triangular nodes depict intracellular and nuclear proteins respectively, diamond nodes are the membrane proteins and square nodes are the proteins with the GO compartment term “unknown”.

By this decomposition method, four successive groups of most active proteins having a role in the information flow were extracted, yielding a key protein subset of approximately 20–30 proteins for each input-output pair. The assembly of these subsets resulted in a smaller but denser “heart” network having 92 nodes and 477 interactions, depicting the most frequent information flow routes for each input-output pair (more than 50% of the linear paths were covered for each pair in the final “heart” network), generated with Cerebral plugin of Cytoscape [97] (Figure 3). The same decomposition method was also applied to the linear path spectrum of Tor1p-Sir2p pair in BioGrid network, and 107 step-specific key proteins emerged for this pair solely, 74 of them absent in the current “heart” network. Moreover, these key proteins did not include almost one fourth of the key proteins determined for Tor1p-Sir2p pair in tCAN (Table S11). Although the number of step-specific key proteins increases approximately five fold in BioGrid network for Tor1p-Sir2p pair compared to tCAN, analysis on BioGrid network fails to detect all of the proteins determined in tCAN. For example, proteins such as Sac6p, Slt2p and Pma1p, which are involved in chronological aging [79], [98], [99] process of *S. cerevisiae* and fission yeast, are not captured in the results of BioGrid Network, but of tCAN. This result implies that the reconstructed network provides a smaller yet more distinctive subset of proteins for chronological aging in *S. cerevisiae*.

The “heart” network (comprised of the key proteins) also includes 19 of the 20 hub proteins given in Figure 2, except Yra1p. This result is actually expected, since hub proteins, having higher connectivity values compared to other proteins, are indeed proved to be active in the information flow in a network [100]. Apart from the 19 hub proteins, several key proteins of the heart network have been reported either to be involved in regulation of chronological life span or to be associated with quiescence in yeast. *BCK1, FMP48, SNF1, TOR1, CKA1* and *RIM15* are among the signal transduction genes whose expression values have been demonstrated to be significantly higher in quiescent cells [101]. Similarly, the deletion mutants of *BCK1, CKA2, UFD2, DFM1, SSD1* and *OSH6* have recently been reported to be among the outgrowing strains in a competition experiment on life span regulation [80]. Overexpression of *ADH1*, which encodes the alcohol dehydrogenase, another key protein in the heart network, resulted in the extension of chronological life span [102]. More importantly, when GO process terms of these 92 key proteins are investigated globally, it is noticed that they overlap with the GO enrichment results attributed to modules of tCAN (Table S12), indicating that the sub network they formed is successful in reflecting the characteristics of tCAN. Moreover, the step-specific key proteins comprising the “heart” network also include the “important” proteins obtained via global participation percentage method.

### Determining New Proteins Regulating the Chronological Life Span (CLS) of Yeast

The proteins in one of the clusters of the heart network (cluster 19 in Figure 3) are of particular interest for further analysis since these four proteins, Tcb3p, Sna3p, Pst2p and YGR130Cp, do not have a known biological process term. Therefore, these key proteins are taken as good candidates to check the validity of the assumption that the members of the heart network are actively contributing to the chronological aging process in yeast, and chronological life span determination experiments were carried out as described in Materials and Methods section with deletion mutants of genes encoding these proteins along with Δras2 strain. The deletion of *RAS2* is known to extend the chronological life span [4]; hence this strain is used as a reference to compare the extent of gene deletion effect on life span.

**Figure 4:**
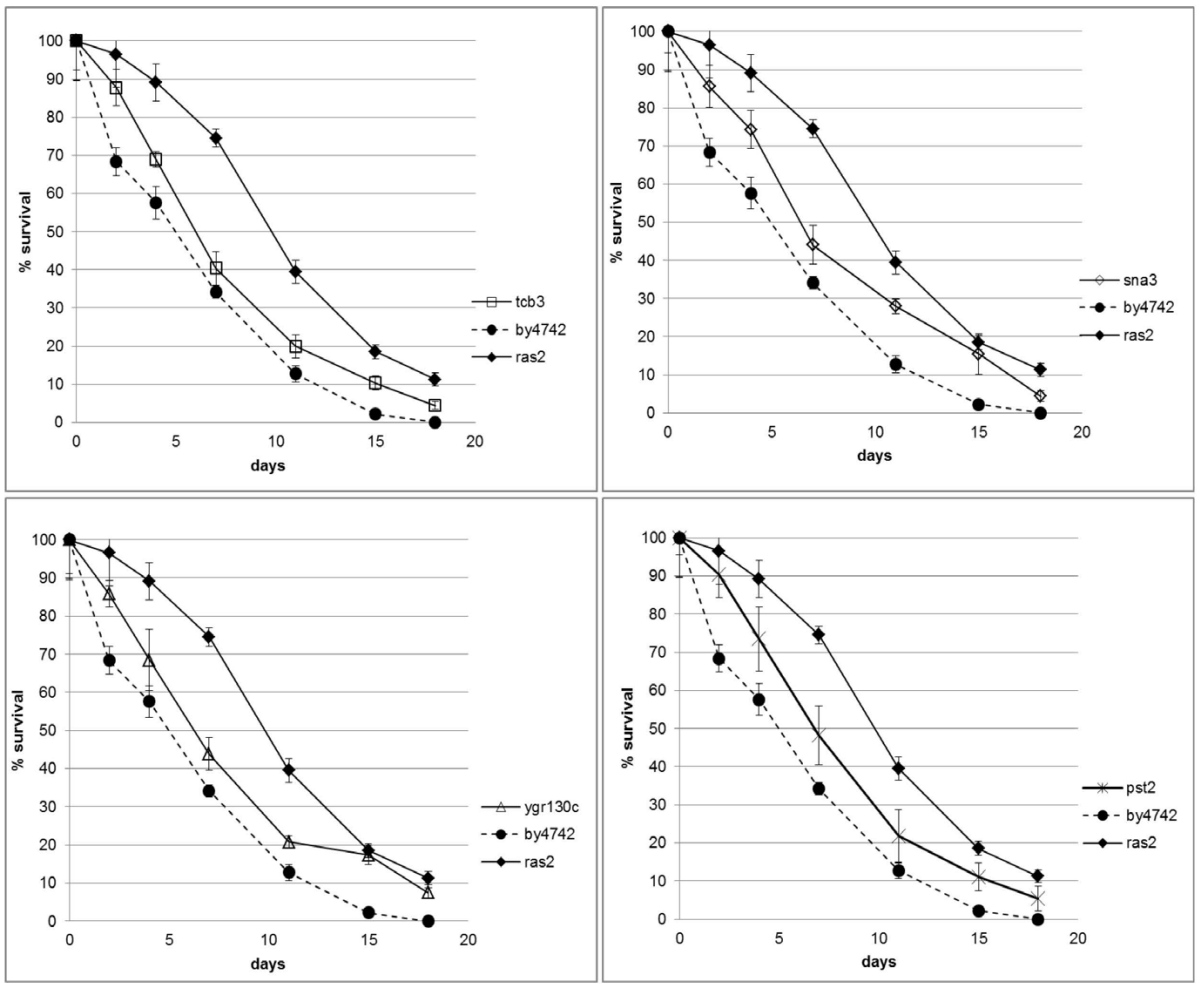
Survival curves of the deletion mutants and wild type strains. The percentages are the average values of 4 experiments (two biological along with their two technical replicates) and error bars denote the standard deviation of the indicated sample.

When the survival curves belonging to these strains are observed, it is noticed that chronological life span was increased for all mutants compared to wild type strain (Figure 4), although the increase was not as pronounced as it is in the case of *RAS2* deletion. This difference is in fact more clearly observed in terms of mean (the day on which survival reaches 50%) and maximum (the day on which the survival reaches 10%) life spans (Table 3).

**Table 3:** Percent increase and p–values of maximum and mean life spans of deletion mutants compared to wild type strain.

	maxlife span	meanlife span	p-value(max life span)	p-value (mean life span)
**Δtcb3**	25	18	5.58E-04	3.55E-03
**Δsna3**	42	27	1.52E-05	2.84E-02
**Δygr130c**	50	18	2.68E-06	1.05E-03
**Δpst2**	25	36	1.79E-02	4.42E-03
**Δras2**	58	82	1.11E-04	1.24E-05

Parallel to our findings, Pst2p and YGR130Cp have recently been reported to be a part of a longevity network of yeast [103]. Pst2p is a flavodoxin-like protein which plays a role in the stress response of yeast, and rapamycin treatment induces a serious growth defect in the homozygous deletion mutant of *PST2* [104]. As for *YGR130C*, whose expression is approximately doubled following a rapamycin treatment in a study investigating nitrogen assimilation in yeast [105], Pst2p is found to be involved in endocytotic machinery of the cell and speculated to control protein turnover [106], [107]. The other membrane protein affecting CLS, Sna3p, is a multivesicular body cargo protein [108] and is a part of the endosomal network. Moreover, overexpression of *SNA3* results in a more stabilized Tat2p, the high-affinity tryptophan permease which is normally degraded upon nutrient starvation or rapamycin treatment [109]. Finally, Tcb3p which is one of the three yeast tricalbins, is another membrane protein involved in membrane trafficking [110] and deletion of *TCB3* renders cells more resistant to rapamycin treatment [48].

In summary, all these 4 proteins share a role in the intracellular transport, mainly endocytotic pathway, and are responsive to rapamycin treatment in yeast cells. Observing the similar survival profiles of the deletion strains of the genes encoding these proteins, one may speculate that the four key proteins Tcb3p, Sna3p, Pst2p and YGR130Cp, affect chronological life span by altering the endosomal network, since endocytosis and vacuolar protein sorting processes are already known to be involved in the regulation of chronological life span in yeast [78], [80].

## Discussion

In this study, the chronological aging network of *S. cerevisiae* is reconstructed in an automated manner, by integrating protein-protein interaction data available in literature with gene ontology terms. It is observed that the resulting network reflects qualities similar with other biological networks such as a scale-free nature and small world architecture, but the current network also possesses a hierarchical nature, implying a regulatory organization within itself. This result is particularly interesting because it stresses out the presence of an information flow embedded in the network from particular nodes to final ones, in a structured and organized manner, as it is the case with signaling networks. In fact, many signaling pathways have proven to alter the chronological life span of yeast, and the current study integrates most of these pathways into a large and hierarchical network.

Network refining is a necessary measure taken to counteract the automated method adopted, to remove insignificant (to the current context of chronological aging) as well as erroneous protein-protein interaction data. In other words, the tuning enabled the investigation of the chronological aging process of yeast with a ≈30% smaller network, refined from erroneous data. The strong overlapping of the results obtained from both topological and modular analyses of the two networks, CAN and tCAN, demonstrated that indeed tCAN is a more refined and to the point version of the initial CAN. Thus betweenness centrality of the nodes is an accurate measure to decide whether the particular node is important when investigating signaling networks, where the participation of nodes to the information flow is crucial.

Several gene ontology process terms involving cell cycle, protein and ROS homeostasis together with reorganization of metabolism, proven to be related to chronological aging in *S. cerevisiae* emerged from enrichment analysis of the modules of CAN and tCAN, fortifying the hypothesis that the reconstructed networks encompass the multi-faceted mechanisms which impact chronological aging in yeast. Protein ubiquitination/degradation and processes pertinent to mitochondria and peroxisomes especially stand out among the enrichment results, stressing out the strong interplay between protein and ROS homeostasis in the cell when chronological aging is concerned. Processes involved in autophagy and apoptosis, especially endocytotic machinery, are also among the key effectors of chronological aging according to the enrichment analysis.

The in-depth investigation of tCAN is achieved via linear path analysis between selected input-output protein pairs, followed by the identification of “step-specific key proteins” of the linear spectra. The quantitative evaluation of the linear path analysis provided preliminary results about the activity and robustness of the branch starting with a specific input protein in the chronological aging network. These characteristics give hints about the complexity of the machinery by which the mentioned input protein affects the life span; increased complexity may imply that the input protein in question alter aging via a broad range of cellular processes. The identification of step-specific key proteins constitutes the qualitative aspect of the linear path analysis, aiming the detection of proteins whose impact on aging is not obviously noticed otherwise.

In fact, a majority of the members of the “heart” network (which is composed of step-specific key proteins) is actually proved to be involved in life span alteration in studies conducted by different groups, hinting that the computational approach adopted is successful in capturing the global picture. Moreover, 4 genes (*TCB3*, *SNA3*, *PST2* and *YGR130C*) which have not been previously reported to be related to the chronological life span of *S. cerevisiae*, encode the key proteins that are not involved in any known process in the cell. The experimental chronological life span assays reveal that the deletion of the genes resulted in an increase in the life span compared to wild type, albeit the rise is not as significant as in the case of the deletion of *RAS2*. The fact that the four proteins are all responsive to rapamycin treatment and related to the endocytotic machinery of the cell implies that they may affect the chronological life span by modifying the endosomal network of yeast. Further experimental work, such as a whole genome analysis may be conducted to comprehend more thoroughly the underlying reorganization of the cellular machinery of these deletion mutant strains leading to an increased life span.

Due to its multifactorial nature, aging remains one of the most complicated and therefore intriguing phenomena of the cell to investigate. The involvement of various signaling pathways is responsible for its multi-faceted nature, as proved here by modular analysis. The disruption of the collaboration between these pathways is essential for aging and also increases the susceptibility of an organism towards age-related diseases. Therefore, the reconstructed network may not only shed light to chronological aging process itself, but also to the fundamental bottleneck points responsible for the degeneration of the cooperation between these signaling branches, which determine the fate of a cell.

## Materials and Methods

### Network Reconstruction by Selective Permissibility Algorithm (SPA)

**Figure 5:**
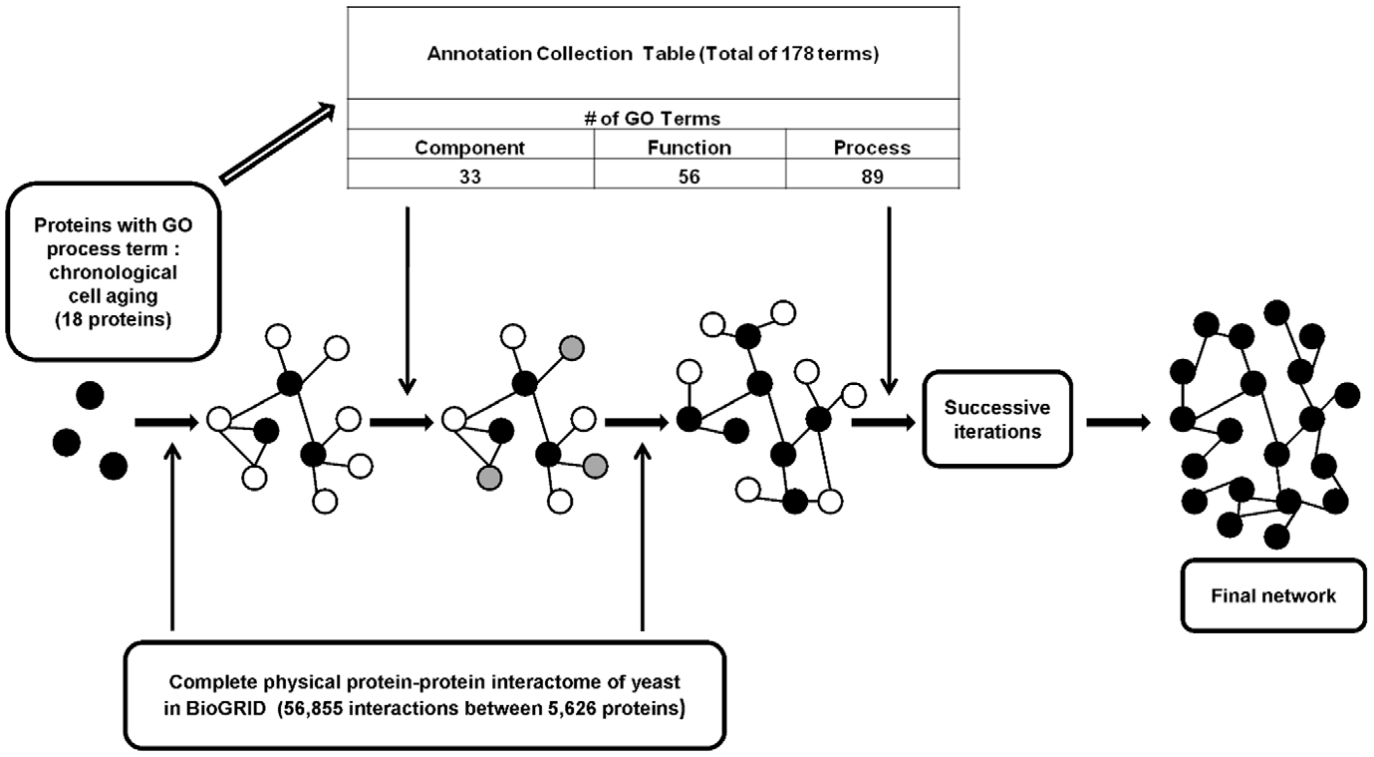
Schematic representation of the network reconstruction algorithm, SPA. The black filled circles represent core proteins, empty circles are possible candidates for first neighbors, and grey filled circles show eliminated candidates via annotation collection table. Process continues on with determination of new candidate proteins as next neighbors based on published PPI data and validation of these candidates by annotation collection table until no new protein is added to the network.

To reconstruct the signaling network of chronological aging in yeast, the Selective Permissibility Algorithm (SPA) was adopted [22]. The inputs of the algorithm were the core proteins of the network and the Annotation Collection Table which is used to expand the network from the mentioned core proteins. To determine the core proteins, gene products which share the “chronological cell aging” GO biological process term were extracted from the manually curated literature data of *Saccharomyces* Genome Database (SGD), released on 29.01.2011 (Table S1). Next, the Annotation Collection table was created by pooling the process, function and component GO annotations of the determined core proteins only. As the third step, all physical interactions of the core protein(s) were extracted from BioGRID database [111] of protein and genetic interactions, release 3.1.73. By integrating GO annotation terms with the interactome data of yeast, the chronological aging network of *S. cerevisiae* is reconstructed. Briefly, a candidate protein was included into the network if all of the three GO annotations (component/function/process) of the protein are present in the Annotation Collection and if it physically interacts with the core proteins, as a first neighbor. Proteins included via this procedure become the “new” input proteins and the algorithm expands the network in this cyclic way until no new interacting proteins are added to the network (Figure 5).

In order to prevent exclusion of proteins solely due to the lack of available literature data, along with the GO annotations of the core proteins, the “biological_process”, “cellular_component” and “molecular_function” terms, namely the “unknown” terms, were also included into the Annotation Collection Table. The Annotation Collection created by this approach covers 178 annotations extracted out of a total of 4,208 annotations (about 4%) (Table S13).

### Network Tuning

The reconstructed network was statistically “tuned” using the betweenness centrality (BC, the number of shortest paths passing through a node (or an edge) given a shortest path algorithm) of a node, which is a measure of a node's importance to the network. It is assumed that the nodes of the reconstructed network should differ significantly in their participation to the information flow, compared to their role in random networks. 100 random networks were generated by shuffling the edges of the original graph, i.e. by preserving the degree of each node. In other words, in the null distribution composed of 100 random networks, all nodes had the same “degree” value as they had in CAN, whereas their interaction partners were randomly selected, opposed to CAN. The original BC value distribution of CAN tended to follow a skewed distribution, hence this trait observed in the distribution was used as a basis in the control chart utilized to check the suitability of the computed null distribution (Figure S4). The randomization procedure and computation of BC values of all nodes (for both the original and randomized networks) are implemented in MATLAB 7.0 (MathWorks, Inc.,Natick, MA) using the MatlabBGL package (written by David Gleich). For randomized networks, average values along with the estimated variances of BC values corresponding to each node are computed and a hypothesis testing is carried out with the following hypotheses:

#### Null Hypothesis

The protein in question is included in the network randomly; its contribution to the putative information flow in the random networks is the same as its contribution to the real information flow of the original network (BC value in CAN is equal to the average BC value of 100 random networks)
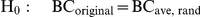



#### Alternative Hypothesis

The contribution of the protein in question to the information flow in the original network differs significantly from that in the random networks (BC value in CAN is not equal to the average BC value of 100 random networks)
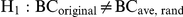



This hypothesis testing is then carried out for all nodes using a dependent, two-tailed t-test for paired samples with a confidence level of 99.9%.

### Determination of Network Topology

Topological properties of both the tuned (reduced) and initial networks, such as degrees, betweenness centralities, diameter, average shortest path length and clustering coefficients were calculated using the algorithm implemented in MATLAB 7.0 (MathWorks, Inc.,Natick, MA) with the MatlabBGL package.

### Cluster Identification and Functional Enrichment

Highly densely connected proteins of the reconstructed networks were identified with MCODE [112] plugin of Cytoscape. In MCODE, loops were included while scoring the networks and the degree cutoff (the minimum degree necessary in order for a node to be scored) value was set to 2. The set cutoff value (the threshold score that determines the inclusion of a node to a cluster, depending on the seed node's score) was 0.2 for cluster expansion; the fluff parameter (the threshold score that determines the inclusion of the neighbors of a node to a cluster, depending on the node's neighborhood density) was turned “off” while the haircut option was “on” (all singly-connected nodes were removed from clusters). Finally, the K-Core value (the minimum degree of a maximally inter-connected sub-cluster within a cluster) was set to 2, and the maximum depth (the distance from the seed node while searching for cluster members) was set to 100. The overrepresented categories within the clusters with an MCODE score greater than 1 or with a node number greater than 3, were further investigated with BINGO plugin of Cytoscape [113]. The enrichment was assessed with the hypergeometric test for the cluster under study versus the whole annotation. The significance level was chosen to be 0.05 and the false discovery rate was controlled with Benjamini and Hochberg correction.

### Identification of Linear Paths and Step-specific Key Proteins

**Figure 6:**
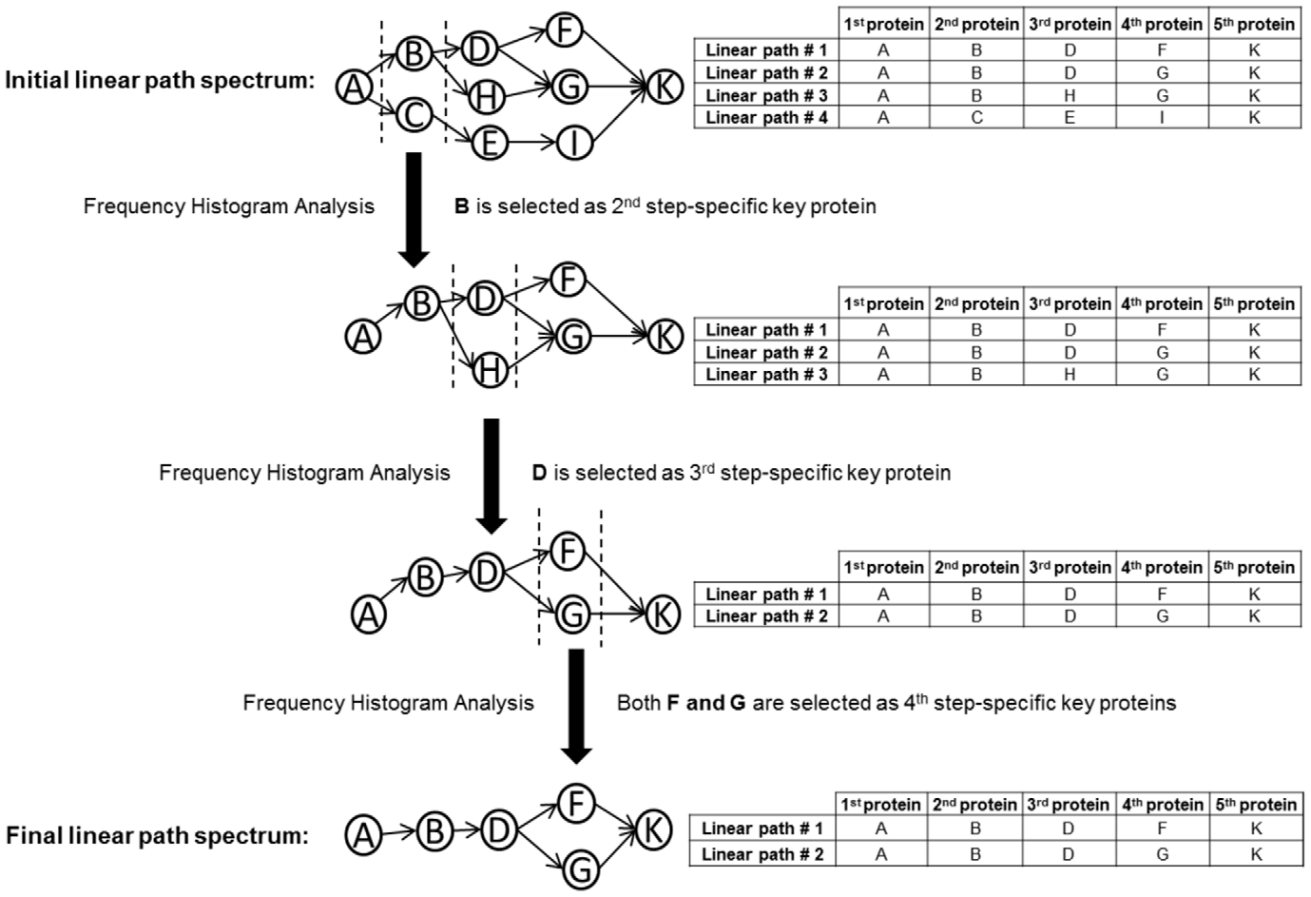
Schematic representation of key protein determination of the linear paths for an input-output pair. The putative input and output proteins are A and K respectively. The procedure is depicted for a path length of 5. For details of the Frequency Histogram Analysis, see Materials and Methods section.

To gain insight on the signal flow in the chronological aging network of *S. cerevisiae*, the linear paths of length 6, between a starting (usually a membrane) protein and a target protein (a transcriptional regulator) were evaluated using NetSearch algorithm [86]. Step-specific key proteins of the linear paths of an input-output protein pair were then determined via decomposing the linear paths into their steps (Figure 6). Briefly, for the first one of the successive 5 steps constituting a linear path of length 6, the percent participation values (ratio of the number of linear paths in which the mentioned protein is present over the total number of linear paths) of involved proteins were calculated. Then a cumulative histogram representing the frequency distribution of these percentage values was created. *m_i_*, the histogram function, counts the number of proteins that fall into each of the disjoint categories (known as “bins”) of participation frequencies. The cumulative histogram *M_i_*, counts the cumulative number of proteins in all of the frequency bins up to the specified bin, represented by eqn. 1: 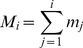
(1) where i is from 1 to k (the square root of the number of proteins participating to the histogram, the bin number). The fold change in the percent difference of the cumulative frequency for each bin is denoted by eqn. 2:
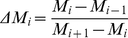
(2) from i = 1 to k-1, with *M*_0_ = 0 and *M_k_* = 100. The i^th^ frequency bin in which the largest percent fold change occurred in frequency was chosen as the threshold bin, if the number of proteins having frequency values larger than that threshold did not exceed 10% of the total number of proteins under study. Otherwise, the frequency bin having the next greater fold change is determined as the threshold. Ultimately, proteins which have frequency values larger than that of the threshold frequency bin are determined as the key proteins specific to the first step. For the successive step, these key proteins were used as “baits”: linear paths involving these proteins as the second proteins were selected and the procedure was repeated to this reduced subset of linear paths to yield “hits”, the key proteins of the third step. Via this decomposition analysis, 4 sets of key proteins (specific to the 1^st^, 2^nd^, 3^rd^ and 4^th^ steps) were determined for each input-output pair in the study. When these sets were pooled and united with the input and output proteins, a relatively smaller subset of proteins describing the whole linear spectra of the 12 branches was obtained (Figure 3).

### Chronological Life Span Assay

All of the experiments were carried out in liquid synthetic dextrose complete (SDC) medium [114] with 2% glucose. The deletion strains used in the study (Table 4) were derived from BY4742. Overnight cultures grown in SDC, were diluted to an OD_600_ value of 0.1, and inoculated into 15 ml centrifuge tubes with a 3 ml fresh SDC medium, maintaining a volume ratio of 1∶5. The cultures were then incubated at 30°C and 180 rpm. Serial dilutions of the culture were spread onto four YPD plates (two biological and two technical replicates) for each strain and time point. Colony formation was monitored after two days. All cultures were presumed to be 100% viable at day 3, with subsequent colony forming unit (CFU) measurements normalized to CFUs of day 3 to obtain survival data.

**Table 4:** Yeast strains used in the study.

Strain	Genotype	Source
BY4742	Mat α; his3Δ1; leu2Δ0; lys2Δ0; ura3Δ0	EUROSCARF
*ΔSNA3*	Mat α; his3Δ1; leu2Δ0; lys2Δ0; ura3Δ0; YJL151c::kanMX4	EUROSCARF
*ΔTCB3*	Mat α; his3Δ1; leu2Δ0; lys2Δ0; ura3Δ0; YML072c::kanMX4	EUROSCARF
*ΔYGR130C*	Mat α; his3Δ1; leu2Δ0; lys2Δ0; ura3Δ0; YGR130c::kanMX4	EUROSCARF
*ΔPST2*	Mat α; his3Δ1; leu2Δ0; lys2Δ0; ura3Δ0; YDR032c::kanMX4	EUROSCARF

**Figure 7:**
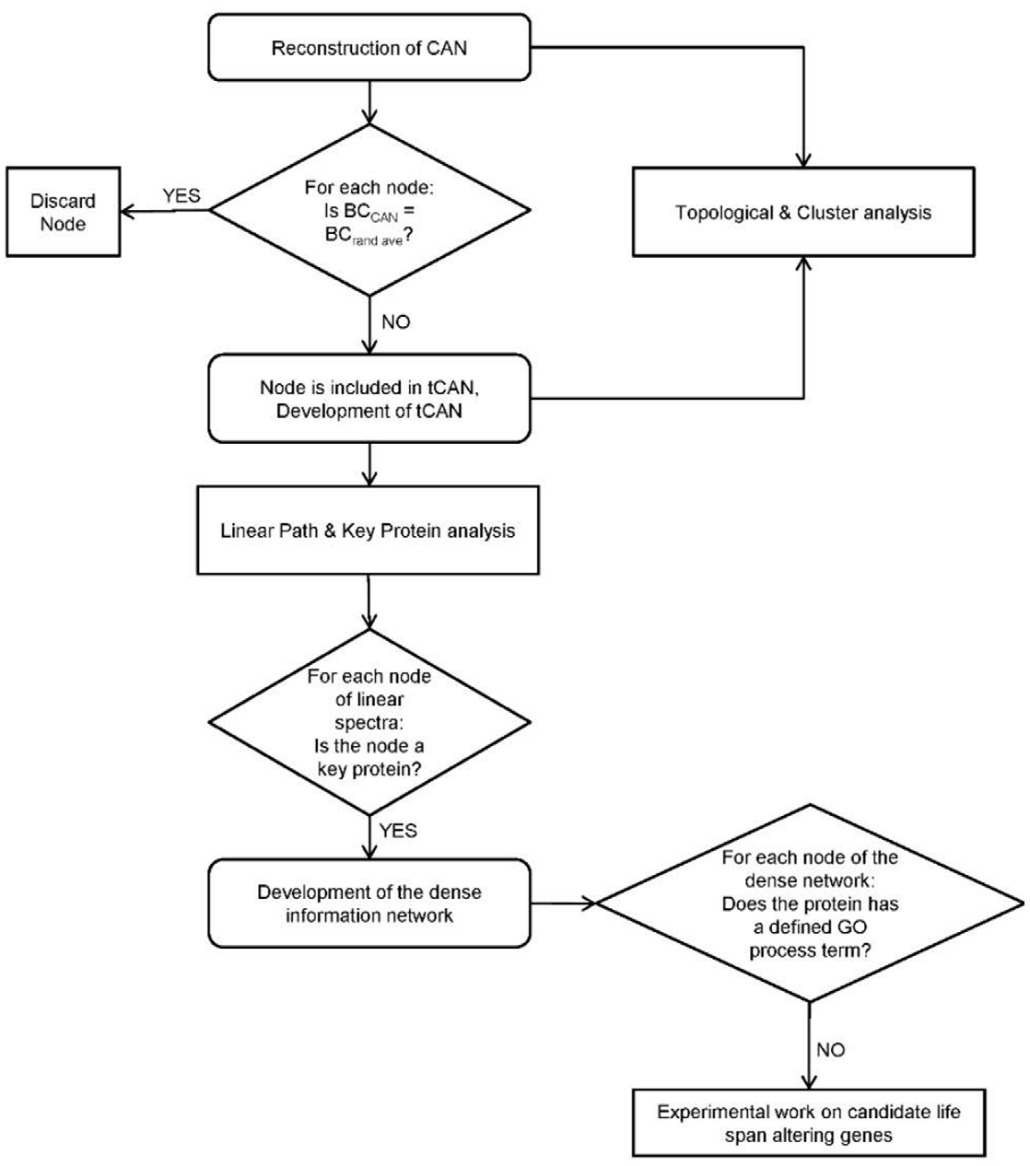
Algorithm of the methodology followed in this work.

The methodology followed in the present work is summarized in Figure 7.

## Supporting Information

Figure S1 **Number of proteins included in the network at each step during expansion.**
(TIFF)

Figure S2 **Topological analysis of BioGrid Network: a) Connectivity and b) average clustering coefficient distributions.**
(TIF)

Figure S3 **Connectivity distributions of the resulting tCAN for a) α = 0.1 and b) α = 0.01.**
(TIF)

Figure S4 **Control chart for the 100 random networks computed.** The chart is based on the skewness property of BC value distribution of the original CAN. CL, UCL (CL + 3σ) and LCL (CL-3σ) correspond to center line, upper and lower control limits respectively.
(TIF)

Table S1 **Core proteins of the network.**
(XLS)

Table S2 **Protein-protein interactions of CAN.**
(XLS)

Table S3 **Protein-protein interactions of tCAN.**
(XLS)

Table S4 **Connected Component (CC) Analysis for the first 20 hubs of CAN, in CAN and BioGrid Network.**
(XLS)

Table S5 **MCODE results of CAN.**
(XLS)

Table S6 **MCODE results of tCAN.**
(XLS)

Table S7 **MCODE results of BioGrid Network.**
(XLS)

Table S8 **BINGO results for the first 6 clusters of BioGrid Network.**
(XLS)

Table S9 **BINGO results for clusters of CAN.**
(XLS)

Table S10 **BINGO results for clusters of tCAN.**
(XLS)

Table S11 **Comparison of the step-specific key proteins of Tor1p-Sir2p pair for tCAN and BioGrid Network.**
(XLS)

Table S12 **GO process terms of the clusters of the "heart" network.**
(XLS)

Table S13 **Annotation Collection Table of the core proteins.**
(XLS)
