# Supplementary figures and images for: A Network-Based Approach on Elucidating the Multi-Faceted Nature of Chronological Aging in S. cerevisiae
Source: PLoS One. 2011 Dec 21;6(12):e29284. doi: 10.1371/journal.pone.0029284 (PMC3244448; doi:10.1371/journal.pone.0029284)

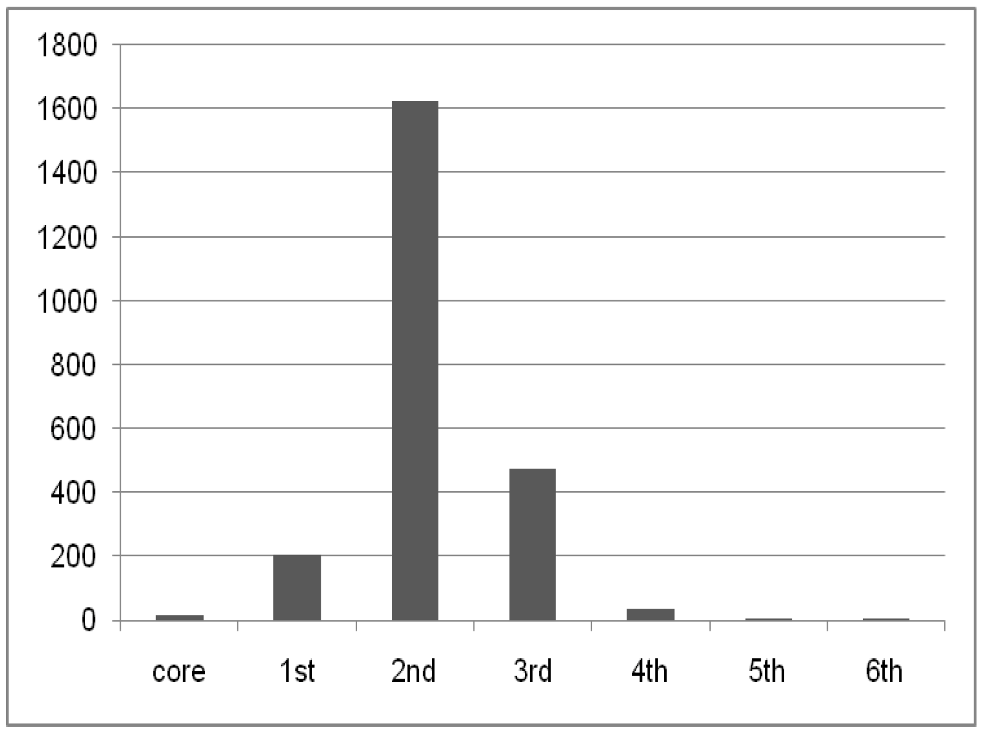

Supplement: Figure S1 — Number of proteins included in the network at each step during expansion. (TIFF) [file pone.0029284.s001.tif]

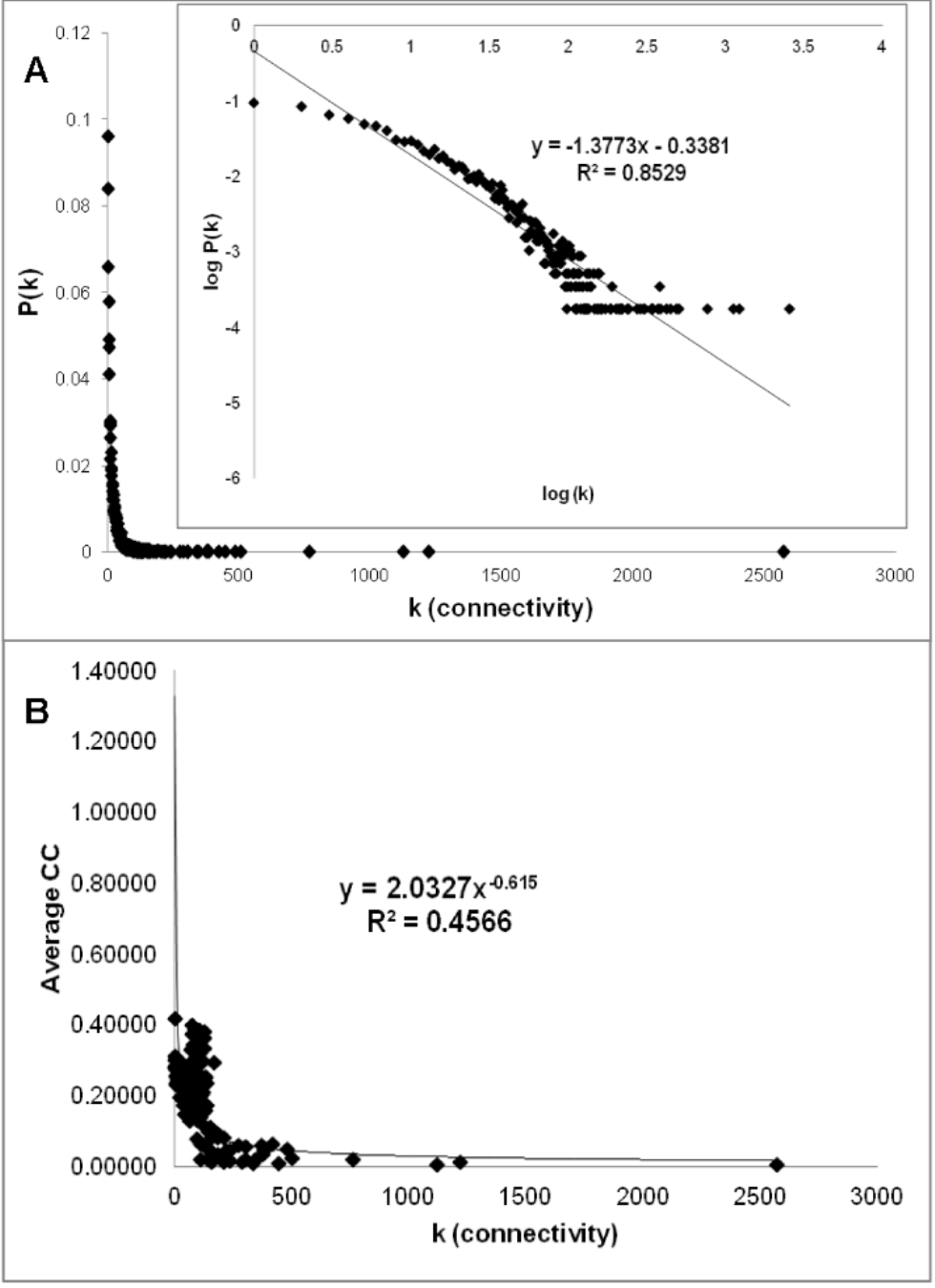

Supplement: Figure S2 — Topological analysis of BioGrid Network: a) Connectivity and b) average clustering coefficient distributions. (TIF) [file pone.0029284.s002.tif]

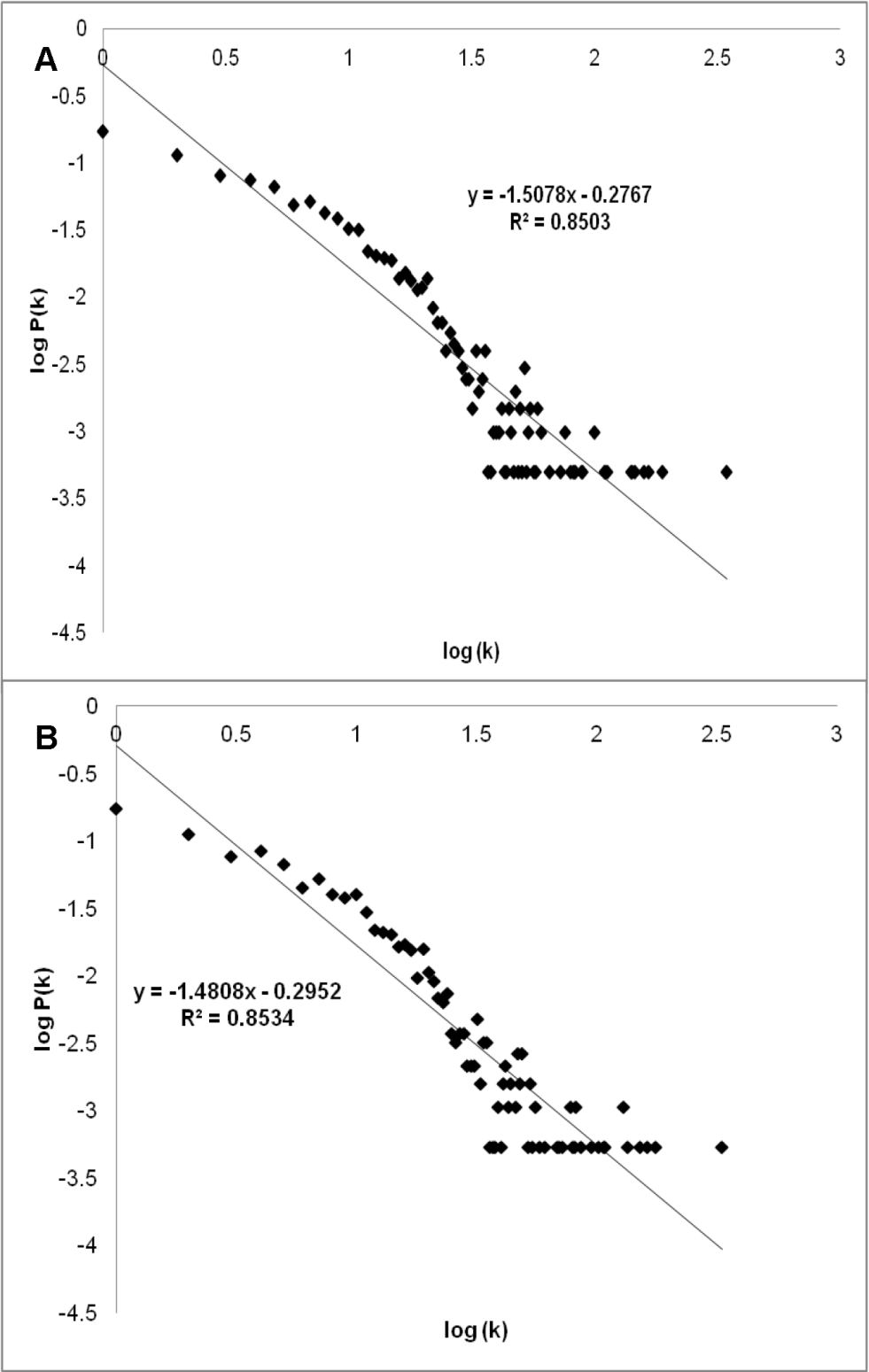

Supplement: Figure S3 — Connectivity distributions of the resulting tCAN for a) α = 0.1 and b) α = 0.01. (TIF) [file pone.0029284.s003.tif]

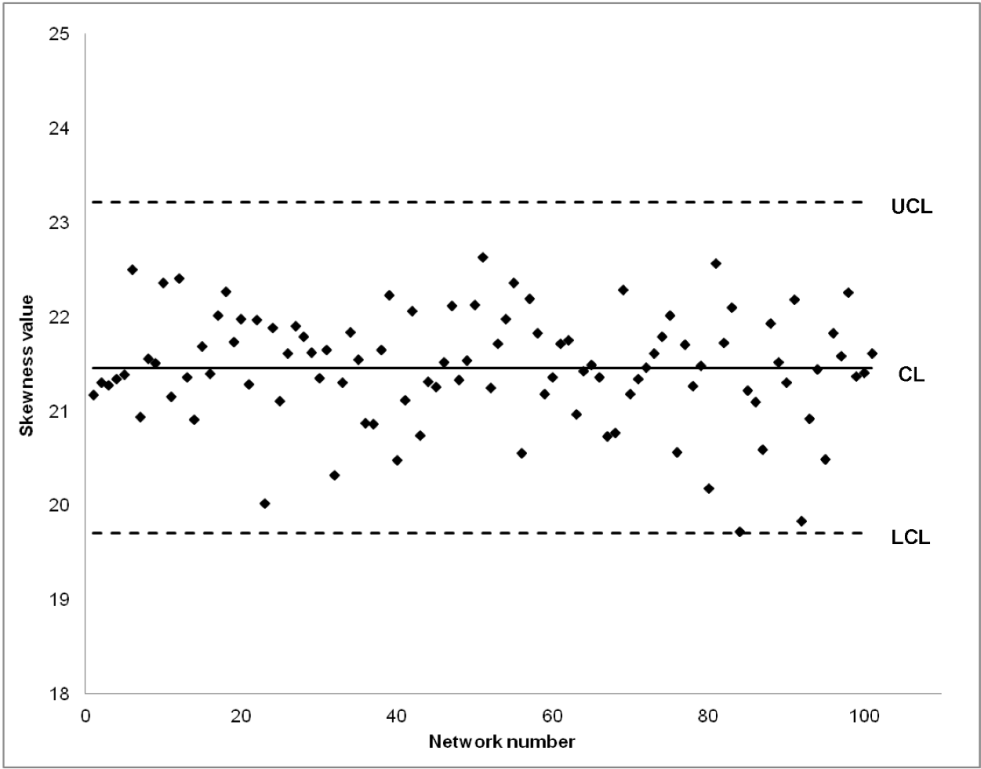

Supplement: Figure S4 — Control chart for the 100 random networks computed. The chart is based on the skewness property of BC value distribution of the original CAN. CL, UCL (CL + 3σ) and LCL (CL-3σ) correspond to center line, upper and lower control limits respectively. (TIF) [file pone.0029284.s004.tif]
